# Facile Use of Silver Nanoparticles-Loaded Alumina/Silica in Nanofluid Formulations for Enhanced Catalytic Performance toward 4-Nitrophenol Reduction

**DOI:** 10.3390/ijerph18062994

**Published:** 2021-03-15

**Authors:** Rashmi Mannu, Vaithinathan Karthikeyan, Murugendrappa Malalkere Veerappa, Vellaisamy A. L. Roy, Anantha-Iyengar Gopalan, Gopalan Saianand, Prashant Sonar, Binrui Xu, Kwang-Pill Lee, Wha-Jung Kim, Dong-Eun Lee, Venkatramanan Kannan

**Affiliations:** 1 Department of Physics, SCSVMV Deemed University, Kanchipuram 631561, India; mrashme@gmail.com; 2 Department of Physics, St. Joseph’s College of Arts and Science for Women, Hosur 635126, India; 3 Department of Materials Science & Engineering, City University of Hong Kong, Hong Kong; kvecers@gmail.com; 4 Department of Physics, BMS College of Engineering, Bangalore 560019, India; murugendrappamv.phy@bmsce.ac.in; 5 Department of Electronics & Nanoscale Engineering, University of Glasgow, Glasgow G12 8QQ, UK; roy.vellaisamy@glasgow.ac.uk; 6 Daeyong Regional Infrastructure Technology Development Center, Kyungpook National University, Daegu 41556, Korea; algopal99@gmail.com (A.-I.G.); kplee@knu.ac.kr (K.-P.L.); kimwj@knu.ac.kr (W.-J.K.); 7 Intelligent Construction Automation Center, Kyungpook National University, Global Plaza 904, Daegu 41566, Korea; 8 Global Center for Environmental Remediation, College of Engineering, Science and Environment, The University of Newcastle, Callaghan, NSW 2308, Australia; SaiAnand.Gopalan@newcastle.edu.au; 9 School of Chemistry and Physics, Queensland University of Technology (QUT), 2 George Street, Brisbane, QLD 4001, Australia; Sonar.Prashant@qut.edu.au; 10 Electrical Engineering College, Henan University of Science and Technology, Box 60, 263 Kaiyuan-Road, Luolong, Luoyang 471023, China; 9906065@haust.edu.cn; 11 School of Architecture, Civil, Environment and Energy Engineering, Kyungpook National University, 80, Daehakro, Buk-gu, Daegu 41566, Korea

**Keywords:** nanofluids, metal oxides, silver nanoparticles, thermo-physical properties, catalytic reduction, 4-nitrophenol

## Abstract

The introduction of toxic chemicals into the environment can result in water pollution leading to the degradation of biodiversity as well as human health. This study presents a new approach of using metal oxides (Al_2_O_3_ and SiO_2_) modified with a plasmonic metal (silver, Ag) nanoparticles (NPs)-based nanofluid (NF) formulation for environmental remediation purposes. Firstly, we prepared the Al_2_O_3_ and SiO_2_ NFs of different concentrations (0.2 to 2.0 weight %) by ultrasonic-assisted dispersion of Al_2_O_3_ and SiO_2_ NPs with water as the base fluid. The thermo-physical (viscosity, activation energy, and thermal conductivity), electrical (AC conductivity and dielectric constant) and physical (ultrasonic velocity, density, refractive index) and stability characteristics were comparatively evaluated. The Al_2_O_3_ and SiO_2_ NPs were then catalytically activated by loading silver NPs to obtain Al_2_O_3_/SiO_2_@Ag composite NPs. The catalytic reduction of 4-nitrophenol (4-NP) with Al_2_O_3_/SiO_2_@Ag based NFs was followed. The catalytic efficiency of Al_2_O_3_@Ag NF and SiO_2_@Ag NF, for the 4-NP catalysis, is compared. Based on the catalytic rate constant evaluation, the catalytic reduction efficiency for 4-NP is found to be superior for 2% weight Al_2_O_3_@Ag NF (92.9 × 10^−3^ s^−1^) as compared to the SiO_2_@Ag NF (29.3 × 10^−3^ s^−1^). Importantly, the enhanced catalytic efficiency of 2% weight Al_2_O_3_@Ag NF for 4-NP removal is much higher than other metal NPs based catalysts reported in the literature, signifying the importance of NF formulation-based catalysis.

## 1. Introduction

In recent years, there is a high demand for a high standard of living conditions with minimized or negligible impact from environmental pollution, which necessitates the development of novel materials or methodologies for treating contaminated media and for environmental remediation [1]. The use of metal/metal oxide nanoparticles (NPs) offers several advantages in water treatment and contaminant removal due to their inherent characteristics such as a high surface area to volume ratio, smaller size, availability of a large number of reactive sites and high capacity for regeneration [2,3,4]. The term nanofluid (NF) refers to a blend comprising NPs of a typical size lesser than 100 nm and a base fluid [5], which is developed based on the concept of dissolving solids in fluids [6]. Earlier studies by Ahuja in 1975, Liu et al. in 1988 and investigators at Argonne National Laboratory (ANL) in 1992, clearly documented that NF is a medium in which NPs are dispersed in fluids [7]. The clear advantage of NF has been documented from the 300 times increase in thermal conductivity of the copper particles dispersed water medium over the base fluid [6]. This triggered research studies on the preparation of metal dispersed NF and applied it in numerous heat transfer applications. Besides, NFs have also shown improved rheological and mass transfer properties [8,9]. Further extension of research activities has been projected on metal oxide-based NFs. 

Aluminum oxide (Al_2_O_3_ or alumina) NPs, with their large surface area and pore-size distributions, are known to possess superior catalytic activities for diverse organic reactions [10]. Several studies have been led to investigate the thermal properties of Al_2_O_3_ NF [11,12]. The significance of alumina in NF formulation has been detailed [13]. The heat and mass transfer effects through a vertical channel upon flowing alumina/water NF have been demonstrated [14]. Thermoelectric performance experiments were performed on power generation facilities and evaluated the microgeneration and heat transfer capabilities of alumina NF in the form of electrolytes. Thermal conductivity enhancement was witnessed for water-based aluminum/aluminum oxide NFs [15]. In another report, details on the Al_2_O_3_ NPs based NFs with pure water, pure ethylene glycol, and water-ethylene glycol mixture, as base fluids are presented in relevance to the hydrodynamic and thermal analysis of turbulent forced-convection flows [16]. Al_2_O_3_ NPs possess the capabilities to function as a heterogeneous Lewis acid catalyst or catalyst support and progress on various catalytic reactions is reviewed [17]. Al_2_O_3_ NPs with large surface area and pore-size distribution, have superior catalytic activities for diverse organic reactions [18]. Metal NPs supported on alumina exhibited excellent catalytic properties [19,20].

The studies on the evaluation of thermal conductivity of SiO_2_/water NF were carried out by Yan et al. [21] with SiO_2_ NPs in the NF having weight ratios like 1.0%, 3.0% and 5.0%, respectively and over a range of temperatures. The thermal conductivity increases with the increase in both temperature and mass fraction and the thermal conductivity increase was correlated to in terms of frequency of particle collision and energy transfer rate. In addition to thermal conductivity, the electrical conductivity of the NF is also measured for different volume fractions at different temperatures. Many studies have been conducted on the preparation of SiO_2_ NF [22,23]. Yan et al. studied the thermophysical characteristic of SiO_2_/water NF and its heat transfer enhancement with the field synergy principle [24]. Studies on heat- and mass-transport in aqueous silica NF revealed that the solvent diffusion coefficient decreases with mass proportions of the NP in the NF [25]. The large mass transfer areas and high solubility were notified and these factors resulted in a 4 times increase in the capacity coefficient of CO_2_ for silica NF than the base fluid (water) without NPs [26]. A study on silica NF was performed to understand the flow and heat transfer behavior in confined environments (microchannels) [27]. The silica NF in a toluene–acetone–water ternary mixture was subjected through a membrane-based micro-contactor at various volumetric flow rates and the results revealed that the enhanced mass transfer arises from Brownian motion of nanoparticles and induced micro-convection [28]. 

Looking through the literature one can see that the properties such as heat transfer, mass transfer, solvent diffusion are strongly influenced by the NF formulation of the metal oxide NPs. Solvent diffusion is normally the rate-limiting step in many industrial processes like drying and related events. Mass transfer effects in NF have been reviewed [29,30]. Mass transfer plays a significant effect on the rate of conversion and product formation in heterogeneous catalytic systems. In particular, if one considers adsorption, surface reaction and desorption as sequential steps in heterogeneous catalytic reactions, the rate of mass transfer to the reactive surface (intraparticle diffusion) in the steady-state are related to the rate of the reaction. Temperature, viscosity and the particle size of the catalyst are expected to influence the reaction rate of the catalytic reaction. Upon considering the various applications of heterogeneous catalysis, catalysis by metal oxides plays a dominant role because metal oxide catalysis covers a large class of catalytic processes [31]. The metal oxide catalyst families used in industrial and academic catalytic applications include silica, alumina, etc. In a recent review, the major industrial applications of supported and unsupported metal oxide catalysts are detailed [32]. Particularly, because silica is “generally recognized as safe” by the US Food and Drug Administration) and it is often considered as a safe catalytic material for environmental remediation [33]. 

Keeping in view that metal oxide NPs are not effective enough when applied in their pure or pristine forms for catalytic applications because of the limited sites for adsorption and further sequential processes in the catalytic reactions, surface modification strategies of metal oxide NPs were explored [34,35,36]. Depending on the catalytic reactions to be applied, the metal oxide NPs can be functionalized with organic moieties or decorated with catalytically effective other metal NPs [37,38,39] and catalytic properties of silver nanoparticles (Ag NPs) supported on silica spheres were evaluated. The method of loading Ag NPs on silica spheres has been reported and Ag NPs on silica surface effectively control the flocculation of catalytic particles during a catalytic process in the solution and such a system has been proved to be the successful catalytic system for the reduction of pollutant dyes [40]. A one-pot methodology was adopted to prepare mesoporous silica NPs decorated with Ag NPs [41]. The author’s group of researchers reported a “seed-mediated” strategy for the higher loading of Ag NPs onto the silica surface [42]. However, one should note that the catalytic performances of surface-modified metal oxide NPs dispersed in NF have not been reported so far.

In the present study, Al_2_O_3_ and SiO_2_ NFs were formulated with water as the base fluid and at the first instant, the thermo-physical properties were comparatively evaluated for similar compositions. Importantly, we have modified the Al_2_O_3_ and SiO_2_ NPs for catalytic applications with the loading of Ag NPs, and for the first time, the catalytic role of Ag NPs loaded Al_2_O_3_ and SiO_2_ in NF formulations was evaluated. Towards this purpose, we have selected the reduction of 4-nitrophenol (4NP) in the presence of sodium borohydride as the model reaction to evaluate the catalytic activity. The choice of catalytic model reaction is based on the fact that the 4NP reduction is a well-controlled process without by-product formation and the major product 4-aminophenol does not interfere with the kinetics analysis of the reaction rate by UV–Vis spectroscopy. It must be noted that 4NP is a side product of many industrial processes and is known as a hazardous product. Hence, its removal from the environment is of paramount importance because of its impact on the negative effects on humans as well as animal blood and resultant illnesses. This study presents the first report on an eco-friendly approach for the removal of 4-NP using Ag NPs loaded Al_2_O_3_ and SiO_2_ in NF formulations.

## 2. Materials and Methods

### 2.1. Preparation of Al_2_O_3_ and SiO_2_ NFs

Al_2_O_3_ and SiO_2_ NFs were prepared by dispersing different concentrations (ranging from 0.2% to 2.0% with a phase of 0.2%) of the respective metal oxide Al_2_O_3_ and SiO_2_ NPs (purchased from US Nano Laboratories, Houston, TX, USA) in distilled water under ultrasonication by applying the sonicator at a frequency 24 kHz for 2 h. The temperature was controlled to be at 303 K. The dispersion was stable over a long period. Typically. in the present study Al_2_O_3_ and SiO_2_ NPs were dispersed in distilled water to obtain the respective NFs at 303 K under sonication with a frequency of 24 kHz for about 2 h to ensure effective dispersion of particles. In this study, 0.080 g of NPs is dispersed in 40 mL of distilled water to synthesize NF in 0.2%, 0.160 g of NPs is dispersed in 40 mL of distilled water to produce NF in 0.4%. Like this, Al_2_O_3_ and SiO_2_ NFs were prepared at ten different concentrations (0.2%, 0.4%, 0.6%, 0.8%, 1.0%, 1.2%, 1.4%, 1.6%, 1.8% and 2.0%) in the steps of 0.2%.

### 2.2. Modification of Al_2_O_3_ and SiO_2_ NFs with Ag NPs for Catalytic Application

**Figure 1:**
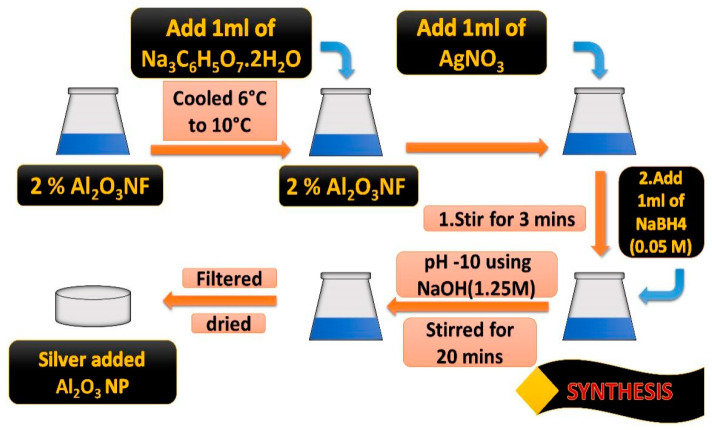
The steps in the preparation of silver-loaded Al_2_O_3_ NPs.

**Scheme 1:**
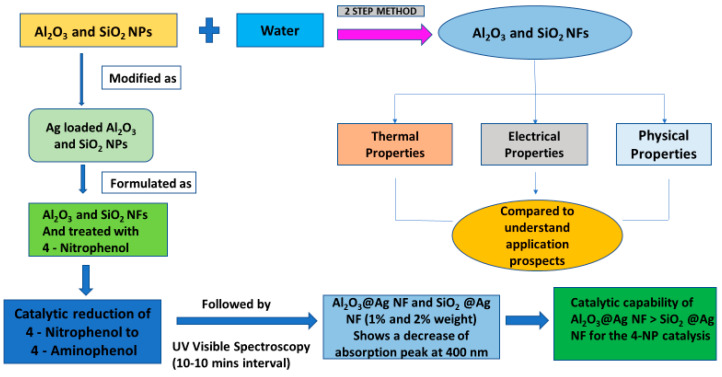
Stages involved in the preparation of nanofluids based on silver encapsulated alumina/silica, evaluation of properties, and catalytic application.

Typically, the procedure for the modification of the respective NFs (Al_2_O_3_/SiO_2_ NFs) with a concentration of 1% NPs in the NF is detailed here. About 1 mL of sodium citrate (0.05 M) and 1 mL of silver nitrate (0.05 M) were added in sequence to the 20 mL NF kept in a cold bath between 6 °C to 10 °C. The solution was stirred for 3 min and subsequently, 1 mL of sodium borohydride (0.05 M) was added slowly. The white-colored solution initial solution turned into light brown. The pH of the solution was changed to 10 by the drop-wise addition of sodium hydroxide (1.25 M) with slow stirring for 20 min and a dark brown colored solution was obtained. The solution was centrifuged, and the supernatant solution was removed. The residue was washed with DI water and dispersed in the NFs with sonication. The details of the procedure are schematically described in Figure 1. The NF prepared with Ag NP loaded Al_2_O_3_/SiO_2_ is designated as Al_2_O_3_(x)@Ag/SiO_2_ (x)@Ag NF, where x refers to the weight % of the respective metal oxide. Scheme 1 shows stages in the formulation of Al_2_O_3_(x)@Ag/SiO_2_ (x)@Ag composite, the properties evaluated and catalytic application of Al_2_O_3_(x)@Ag/SiO_2_ (x)@Ag NF.

### 2.3. Characterization

The characteristics of the Al_2_O_3_ and SiO_2_ NFs such as stability, thermophysical and electrical properties were evaluated by the following experiments. Zetasizer Nano ZS90 (Malvern Instruments Ltd., Malvern, UK, accuracy of 0.12 µm·cm/V·s) was used to measure the Zeta potential of the NFs. Ultrasonic velocity measurements were made using a 2 MHz, F-81 model single frequency ultrasonic interferometer (Mittal, Delhi, India; accuracy ± 0.1 ms^−1^) at 303 K. A digital viscometer (Brookfield, Middleboro, MA, USA; torque: 20.5%, 75 RPM and accuracy ± 0.01 cP) was used to measure the viscosity of all the NFs at four different temperatures (303, 308, 313 and 318 K). The density of the NFs was determined using a specific gravity bottle (5 cc, accuracy ± 0.001 gm). An Abbe refractometer (accuracy ± 1 or 2 units of the 4th decimal place) was used for refractive index measurements. A 6500B precision impedance analyzer (Wayne Kerr, Ghaziabad, UP, India), capacitance, inductance, reactance and impedance basic accuracy ± 0.05%, dissipation factor accuracy ± 0.0005 and quality factor accuracy ± 0.05%) was employed for the electrical measurements at 303 K to a maximum frequency range of 5 MHz. The digital conductivity meter (alpha-06 model, Panchkula, Haryana, India, accuracy ± 1%) was utilized to measure the electrical conductivity at different temperatures (298 K, 303 K, 308 K and 313 K) on a scale of 20 mS. The digital pH meter (alpha-01 model, accuracy + 0.01 pH) was used to find the pH values at 303 K. A GT Sonic Professional Ultrasonic cleaner (Shenzhen, China) is used for sonication, working at the frequency of 40 kHz.

XRD patterns of the Al_2_O_3_ and SiO_2_ NPs were determined using the X’Pert PRO PANalytical powder X-ray diffractometer (Panalytical, Brighton, UK) and the diffraction patterns were recorded at room temperature using Cu Kα radiation (k = 1.5406 å) with the Bragg’s angle varying from 10° to 80°, with a step size of 0.05°. scanning electron microscopy (SEM) is used for analyzing high-resolution surface imaging using a JSM-6390 LV (JEOL, Peabody, MA, USA) attached to an Oxford instruments EDX (Abingdon, UK). High-resolution transmission electron microscopy was measured using JEOL JEM 2100 (Tokyo, Japan). 

## 3. Results and Discussion

The measurements of physicochemical (thermal and electrical), physical (density, refractive index) of Al_2_O_3_/SiO_2_ NFs with varying contents of metal oxide NPs in the base fluid (water) can provide, in particular, the information of the molecular interactions existing between the dispersed NPs, which will form the basis to understand the application prospects. The results and discussion section is divided into two parts: (i) characteristics, stability and properties of Al_2_O_3_/SiO_2_ NFs and (ii) catalytic reduction of 4-NP by Al_2_O_3_(x)@Ag/SiO_2_ (x)@Ag NF.

### 3.1. Al_2_O_3_/SiO_2_ NFs: Characteristics, Stability and Properties

#### 3.1.1. Characteristics and Stability of NFs

##### Characteristics of NFs

**Figure A1:**
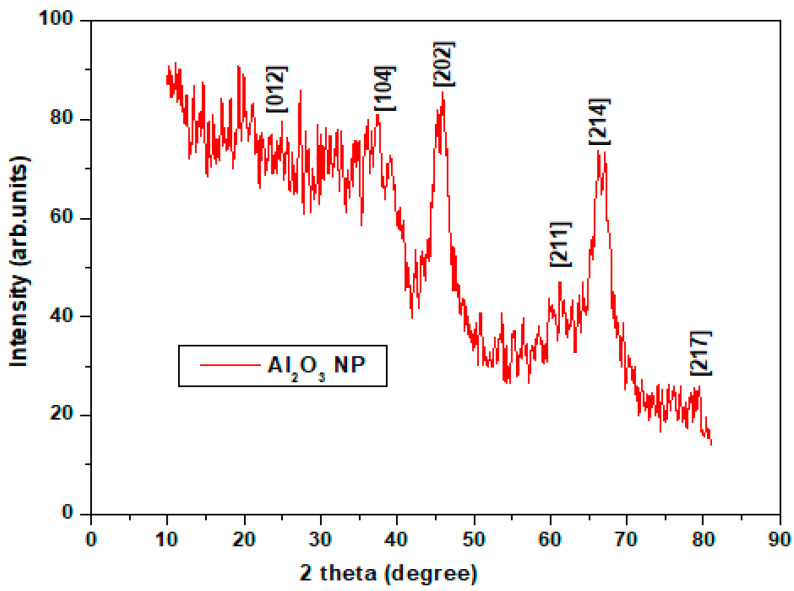
X-ray diffraction pattern of Al_2_O_3_ NPs.

**Figure A2:**
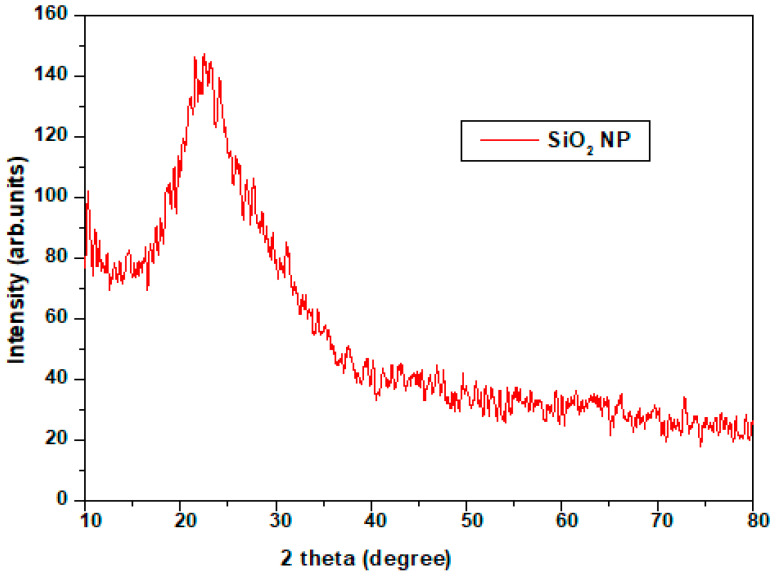
X-ray diffraction pattern of SiO_2_ NPs.

**Figure A3:**
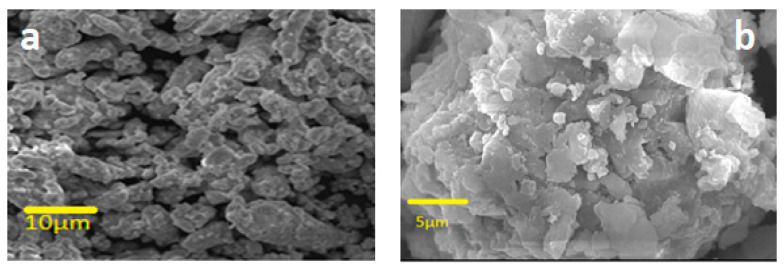
SEM image of (**a**) Al_2_O_3_ NPs and (**b**) SiO_2_ NPs.

**Figure A4:**
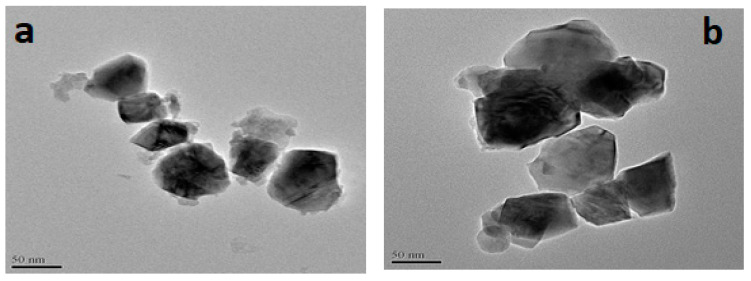
TEM image of (**a**) Al_2_O_3_ NPs and (**b**) SiO_2_ NPs.

**Figure A5:**
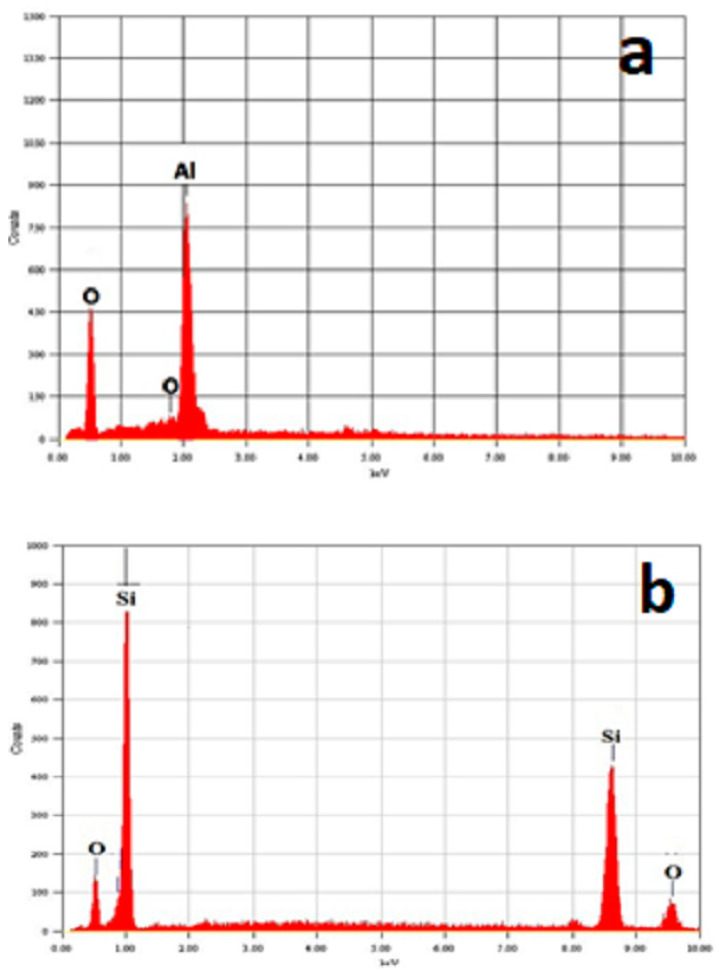
EDS image of (**a**) Al_2_O_3_ NPs and (**b**) SiO_2_ NPs.

**Figure A6:**
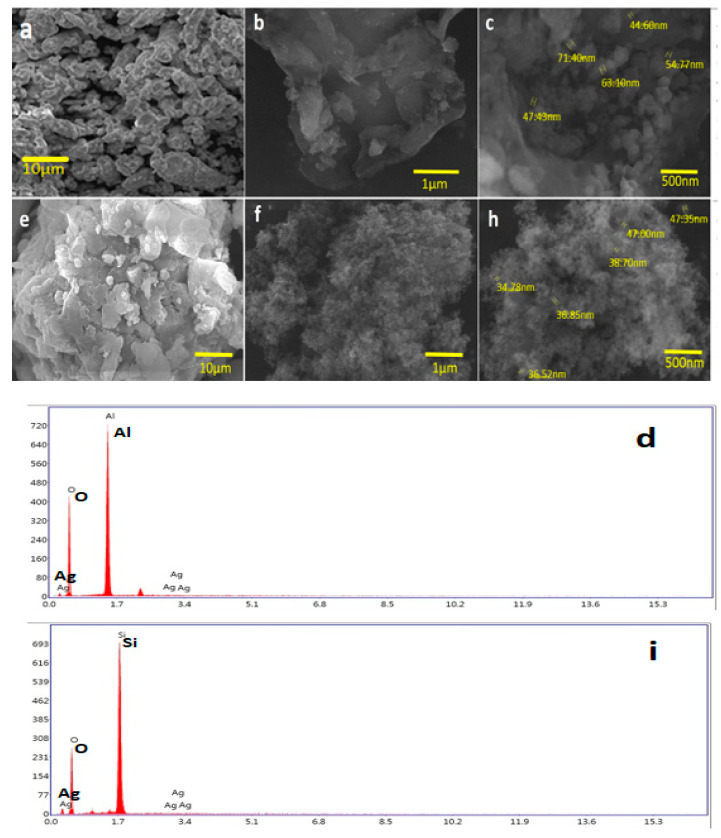
SEM images in different magnifications of (**a**–**c**) Ag loaded Al_2_O_3_ NPs and (**e**,**f**,**h**) Ag loaded SiO_2_ NPs. EDS of (**d**) Ag loaded Al_2_O_3_ NPs and (**i**) Ag loaded SiO_2_ NPs.

The characteristics (morphology and microstructural) as obtained from XRD, SEM, TEM and EDX are presented in Appendix A (Figure A1, Figure A2, Figure A3, Figure A4, Figure A5 and Figure A6) and discussed below (after the Results and Discussion part).

##### Stability of NFs

There are three types of stability: kinetic, dispersion, and chemical stability. When the effective Brownian motion is hindered, the sedimentation of the NPs is facilitated. The stability arising from the amplified movement of the NPs is kinetic stability. The accumulation of the NPs can influence the dispersion and stability arising in the NF as the consequence of dispersion modification is the dispersion stability of the NF. Chemical stability is related to the chemical reaction between the NPs and base fluid. The stability of the NF contrasts with the revolving velocity of the dispersed NPs [43]. Zeta potential is the electric potential in the interfacial double layer at the location of the slipping plane versus a point in bulk fluid away from the interface and it represents the potential difference between the dispersion medium and the stationary layer of fluid attached to the dispersed particle. The significance of zeta potential is that its value can be correlated to the stability of colloidal dispersions. So, colloids with high zeta potential (negative or positive) are electrically stabilized, while colloids with low zeta potentials tend to coagulate or flocculate. The zeta potential values are measured to confirm the stability of the Al_2_O_3_/SiO_2_ NFs, as their stability plays a significant role in applications, especially for the catalysis of chemical reactions. The Zeta potential values measured at 1.0% of concentration are +53.2 mV for Al_2_O_3_ and −1.00 mV for SiO_2_ NF. As a general guideline, colloids with high zeta potential (negative or positive) are considered electrically stabilized. On the other hand, colloids with low zeta potentials may tend to coagulation or flocculation on a long time of standing. Particularly, the NF having a zeta potential value in the range of 40–60 mV is considered to possess good stability based on the electrical potential developed in the NF. Considering the above aspects, Al_2_O_3_ NF with a zeta potential of +53.2 mV is having relatively good stability. The Zeta potential of −1.00 mV for SiO_2_ NF is not in favor of good stability based on electrical double layer development. However, SiO_2_ NF can have chemical stability in relevance to the application. We have modified both Al_2_O_3_ and SiO_2_NPs with Ag NPs towards catalytic reactions. The Zeta potential values of 1% and 2% Al_2_O_3_@Ag NF were −182.0 mV and −200.0 mV, respectively informing the excellent stability for the Ag modified Al_2_O_3_ NF. Similarly, the Zeta potential values of 1% and 2% SiO_2_ @Ag NF were +200.0 mV and +200.0 mV, respectively suggesting that NFs based on Ag modified SiO_2_ were having excellent stabilities. Thus, both Al_2_O_3_@Ag NF and SiO_2_ @Ag NF could be used for following the kinetics of catalytic reactions.

#### 3.1.2. Properties of NFs

The measurements of physicochemical (thermal and electrical), physical (density, refractive index) of Al_2_O_3_/SiO_2_ NFs with varying contents of metal oxide NPs in the base fluid (water) can provide, in particular, the information of the molecular interactions existing between the dispersed NPs, which will form the basis to understand the application prospects.

##### Transport Properties

- Viscosity

Viscosity is one of the important physicochemical properties of pure liquids/liquid-liquid mixtures/liquid-dispersed solid suspensions and it needs to be optimized in many industrial processes. The viscosity arises from the collision among the molecules in the pure liquid or from the interaction between molecules of the liquid and suspended particles in suspension. The force fields operating among the molecules/particles determine the interactions among them. In pure liquids or liquid/dispersed particle suspensions, there can be substantial attractive and cohesive forces between the molecules of a liquid or particles. The cohesion, interactions and molecular interchange can be influenced both by temperature and by the concentration of suspended particles in the case of liquid/suspended particles suspensions [44]. It is known that viscosity plays a major role in the overall performance of a heat transfer fluid. For example, upon pumping of a fluid having increased viscosity through a heat exchanger needs increased pumping energy and therefore reduces the overall benefit of an efficient thermal conductivity for the fluid [45,46]. The influence of increasing the temperature of a liquid is generally regarded to reduce the cohesive forces, on the other hand simultaneously increase the rate of the molecular interchange. The former effect can lead to a decrease in shear stress, but the latter can increase. In short, the viscosity of the fluid is influenced when there is a huge temperature difference or in the presence of particles in suspensions. Relative viscosity (cP) was measured for Al_2_O_3_ and SiO_2_ NFs at four different temperatures (303 K, 308 K, 313 K and 318 K) and shown in Table 1.

It is noticed that viscosity decreases with an increase in temperature due to the dispersion of NPs. The rheology of colloidal dispersion is related to the relative behavior of suspension rather than Newtonian or non-Newtonian in behavior. An inverse relation between viscosity and the linear function of temperature is envisioned in this work [47]. The chief parameters that influence the viscosity of NFs are temperature, NPs volume fraction, size, shape, pH and shearing rate [48]. It is difficult to individually address these factors, rather the synergistic effects can be projected. It is noticed the viscosity of Al_2_O_3_ and SiO_2_ NFs increases in the concentration range from 0.2 to 2.0% and decreases with an increase in temperature. The decrease in viscosity reveals the weakening of intermolecular forces due to the thermal agitation of the molecules. The viscosity of Al_2_O_3_ NF in EG with different ratios of water and EG increased with the increase of particle loading and decreased exponentially with the increase in temperature [49]. It is generally noticed that viscosity-temperature relations are dependent on activation energies for viscous flow. The structural changes taking place in the distributed particles of the NF due to temperature changes are responsible for this phenomenon. In a chemical system, the amount of energy that is required to orient the atoms or molecules for a chemical reaction is known as the pre-exponential factor [47].

- Activation Energy

**Figure 2:**
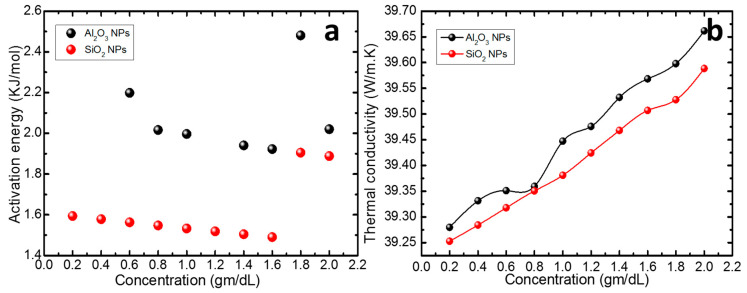
(**a**) Variation of activation energy with concentration (**b**) Variation of thermal conductivity with concentration. In Figure 2b the thermal conductivity data points are joined in terms of concentrations to infer the trend.

It must be noted that viscosity is regarded as the thermally activated process and the requirement is that the molecules/particles need to surmount the activation energy (Q) barrier that arises due to the resistance of the surrounding building units. The value of Q can be calculated from Arrhenius expression given by Moore:

η = A e^Q/RT^ (1)

where Q is the apparent activation energy of flow, and A is the pre-exponential factor. To calculate the Q for the Al_2_O_3_/SiO_2_ NFs, the semi-logarithmic plot between the logarithm of cP and inverse of temperature was used. These plots were linear and the slopes were used to deduce the Q of Al_2_O_3_ and SiO_2_ NFs. Figure 2a presents the plot of Q vs. concentration of NPs in the NF. From Figure 2a, it is understood that the Q of Al_2_O_3_ NFs decreases initially and then increases with a maximum at 0.6 wt% and subsequently shows periodic variations of decrease and increase with the concentration of NFs (>0.6 wt%). Whereas, in the case of SiO_2_ NFs, the Q value gradually decreases up to 1.6 wt%, and thereafter witnessed a shoot up at 1.8% and again slightly decreases at 2.0%. Many reports are available in fluid mechanics to inform that there are relations between Q or A along with the rate of chemical reactions [50,51,52]. The understanding of the kinetics of the chemical reaction about A has significance for various applications that include pollution control, food processing, creation and diffusion of fog, biochemical engineering, catalysis, etc. [53].

- Thermal Conductivity

Thermal conductivity is the primary property among the thermophysical properties of the NFs, which depends on the relevant parameters of NPs material, volume fraction, size, aspect ratio, base fluid, temperature and surfactant. Literature informs that the suspension of solids in fluids enhances the effective thermal conductivity of the material and working fluids with substantial enhanced thermal conductivities that can be used in thermal devices [54]. From the following mathematical expression, the thermal conductivity of the Al_2_O_3_/SiO_2_ NFs is computed:

k = 3 (*N*/*V*)^2/3^ K (2)

where k—thermal conductivity, *V*—velocity of the NFs and *N*—Avogadro’s number. From Figure 2b, it is observed that the k of Al_2_O_3_ NF is higher than SiO_2_ NFs. Few typically reported information is presented here. The k of Al_2_O_3_, MgO, TiO_2_, ZnO and SiO_2_ have been earlier investigated for the NPs having 20 nm NPs dispersed in ethylene glycol [55]. For a volume fraction of 5.0% NPs, the enhancement in k of SiO_2_/ethylene glycol was approximately 25%, while for γ-Al_2_O_3_/EG, the increase in k is approximately 28% [56]. The k values of SiO_2_/ethylene glycol NPs at a temperature of 298.15 showed a linear increase with the increasing volume fraction of NPs [57]. The k values of SiO_2_/water NFs were determined for 1 to 4% volume fractions and the result shows an enhancement in k of 24% at a NPs volume fraction of 4.0% [58].

- Density

The measured values of the density of Al_2_O_3_ NF seem to be low than SiO_2_ NF. But for both cases, it is observed that density increases gradually with an increase in NP. This could be due to the increase in intra-molecular interaction as compared to intermolecular interaction. At 2.0% of concentration the density value is maximum (1008.41 kg/m^3^ for Al_2_O_3_, 1011.78 kg/m^3^ for SiO_2_ NFs).

##### Electrical Properties

- Influence of AC Conductivity and Dielectric Constant

**Figure 3:**
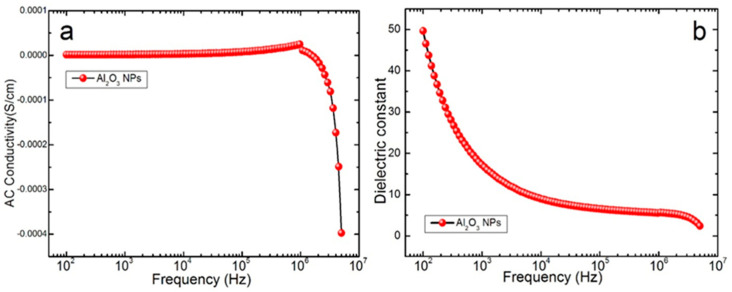
(**a**) Variation of AC conductivity and (**b**) dielectric constant with frequency.

Impedance analyzer is used to find the electrical property of the Al_2_O_3_ NPs at different frequency range over 5 MHz at 303 K. AC conductivity σ_ac_ (ω) is calculated in the frequency range of 100 Hz to 5 MHz using the following equation:

σ_ac_ (ω) = G(ω)t/A (3)

where G is the conductance, t is the thickness of the pellet formed in cm and A is the area of cross-section of pellets of Al_2_O_3_ NPs in cm^2^. The variation of σ_ac_ with frequency at 303 K is presented in Figure 3a. One can notice that σac marginally increases for Al_2_O_3_ NPs up to 1 MHz and then shows a drastic decreasing trend for higher frequencies. For comparative purposes, few of the results reported in the literature are used. The electrical properties of conducting polypyrrole (PPy)–cobalt aluminum oxide (CAO) nanocomposites were studied using an impedance analyzer in the frequency range of 100 Hz to 5 MHz. The dielectric properties and σ_ac_ were determined for the nanocomposites of different compositions in the temperature range of room temperature to 180 °C. The increase in frequency is reflected with a decrease in dielectric constant for all the nanocomposites for the increase in frequency. In a subsequent report, the σ_ac_ values of PPy have been amended for identifying the role of the added CAO nanopowder [59]. Shen et al. reported that σ_ac_ of ZnO NFs is greatly influenced by Brownian motion, agglomeration, and stability of the NF [60]. Zyla and Fal carried out research on the electrical conductivity of a silicon dioxide–glycol nanofluid and understood that when the concentration of NPs in the NF increased, a linear increase in σ_ac_ was witnessed [57]. Our experimental results on the dependence of σ_ac_ with frequency follow the barrier hopping mechanism (CBH) and polarization of dipoles. In this work, a vital assessment of a few of the electrical properties of Al_2_O_3_ in water could be reported. However, the amorphous nature of SiO_2_ NPs restricts the ease of preparation of the sample, and pellet formation was not possible in such a case. The various electrical conductivity parameters were obtained from the impedance analyzer and presented in Table 2. It is found that values of impedance, resistance, D-factor, reactance, and capacitance decrease as the range of frequency increases. But the values of angle increase with an increase in frequency.

Dielectric properties of Al_2_O_3_ NPs were determined in the frequency range between 100 Hz to 5 MHz. The dielectric constant of the system subjected to an external oscillating electric field is calculated by using the formula:

ε’(ω) = Ct/ɛ˳A(4)

where C is the capacitance in Farad, t is the thickness in cm and A is the area of the sample in cm^2^. The frequency dependence of the ε’(ω) at room temperature for Al_2_O_3_ is shown in Figure 3b. It is observed that ε’(ω) is comparatively high at lower frequencies and gradually decreases with an increase in frequency. The different polarization mechanisms like ionic, electronic, orientation, and space charge, which have different relaxation frequencies, could be assigned as the causes for a decrease in ε’(ω) with frequency.

- Influence of Electrical Conductivity and pH of NFs

The electrical conductivity of Al_2_O_3_ and SiO_2_NFs is measured for various concentrations (0.2–2.0%) and temperatures (298, 303, 308 and 313 K), and the results are presented in Table 3. The electrical conductivity of Al_2_O_3_ and SiO_2_ NFs shows an increasing trend with weight fraction (%), however, decreases gradually with temperature. The maximum value of electrical conductivity at 2.0% is 6.06 mS for Al_2_O_3_ NF and 3.56 mS for SiO_2_ NF at 298 K. Table 3 also shows the variation of electrical conductivity with the pH of Al_2_O_3_ and SiO_2_NFs at 303 K.

On perusal of Table 3, one can notice that pH increases gradually and marginally with an increased concentration of the NPs (0.2–2.0%). The maximum pH value of Al_2_O_3_ NF is 8.62 and SiO_2_ NF is 7.03 for 2.0% of concentration at 303 K, which is higher than the pH of base fluid (double distilled water-7.00). It is expected that the electrical conductivity of the NFs is influenced by ionic strength, viscosity, and an electric double layer that is formed all over the particle surface when the NPs are dispersed in a base fluid [61]. The 5.5 times increase in electrical conductivity for Al_2_O_3_ NFs concerning base fluid has been reported in the literature. In the case of SiO_2_-lignin NFs, an increasing trend of electrical conductivity of the NFs with an increase in mass fraction has been observed [62].

#### 3.1.3. Physical Properties

##### Ultrasonic Velocity

The propagation of ultrasound (ultrasound velocity) in a medium is dependent on the NPs concentration. The values of ultrasonic velocity are calculated using the formula [U = N × λ] for the different NFs concentrations under investigation. Table 4 shows the values of ultrasonic velocity, density, and refractive index of Al_2_O_3_ and SiO_2_ NFs at concentrations ranging from 0.2% to 2.0% in weight fraction at 303 K. It is observed that ultrasonic velocity, increases with the concentration of NPs in the NF. The ultrasonic velocity is minimum for 0.2% and maximum for 2.0%. The linear increase of velocity with concentration is seen in both the NFs system. The observed increase of ultrasonic velocity in the Al_2_O_3_ and SiO_2_ NFs is due to the presence of a molecular association between the respective NPs and water molecules [56].

##### Refractive Index

The refractive index (RI) informs the interaction between light and NPs in the NF [63]. RI value is found to be minimum with a value of 1.335 for Al_2_O_3_ NFs in the lower concentration range (0.2%) and 1.337 for SiO_2_ NFs. Then RI value was maximum at higher concentration (2.0%), 1.345 for Al_2_O_3_, and 1.346 for SiO_2_ NFs, respectively. The RI of the NF is increasing linearly with an increase in the concentration of the NPs in the NF.

### 3.2. Catalytic 4-Nitrophenol Reduction by Al_2_O_3_@Ag/SiO_2_@Ag NFs

Water pollution is one of the serious worldwide issues. The 4-NP is regarded as one of the toxic and refractory priority pollutants because it can damage the central nervous system of human beings [64]. 4-NP enters the environment as a chemical pollutant in the form of industrial effluents and agricultural wastewaters. 4-NP is used mainly to manufacture drugs, fungicides, to darken leather, in dyestuff production, and for military purposes. Because of the normal and widespread use, 4-NP can exist as a pollutant in industrial wastewater streams associated with its formulation, distribution, and application. Furthermore, hydrolysis of pesticides and herbicides can also release 4-NP into the subsurface and then contaminate groundwater resources. When the 4-NP is released into the environment, its contagion can cause a noteworthy environmental and public health risk, owing to its delicate toxicity and mutagenic potential. The acute exposure of 4-NP may lead to blood disorders along with methemoglobin. There are different strategies dedicated to 4-NP removal from water. The complete conversion of 4-nitrophenol can also be confirmed by the transparent color change of the solution from initially bright yellow to colorless [65].

The reduction technique involving heterogeneous catalysis is widely used as the cost-effective method for the degradation of many pollutants for the process of removing the pollutants from an aqueous solution. The utilization of physical support for metal catalysts is considered a good choice for designing heterogeneous catalyst systems because it ensures optimal performance and minimal cost for the primary catalyst—active metal phase –through adequate dispersion of metal particles over appropriate support. Such a heterogeneous catalyst system can provide adequate stability for the metal NPs. Typically, direct hydrogenation of 4-NP to 4-aminophenol (a lesser toxic chemical) by the use of a stable heterogeneous catalyst in the presence of a reducing reagent is considered as the beneficial approach to remediate 4-NP. The reduction processes involved in the conversion 4-NP to 4- aminophenol by heterogeneous catalysis are expected to be influenced by several factors such as stability of the catalyst particles in the dispersion state, viscosity activity coefficient, activation energy, effective mass (density), dielectric constant, diffusion coefficient, electric field, current density (charge transfer resistance), charge/electron transfer coefficient (impedance), Zeta potential, pH of the medium, etc. Complete knowledge about the influence of all of these parameters involved in the heterogeneous catalytic processes will be useful for optimization towards achieving the best overall catalytic reduction efficiency from the NFs. Of course, optimizing all these parameters is a time-consuming and tedious task. Alternatively, the quantitative understanding of at least a few of the associated physical and chemical properties of the catalyst system in the NF formulation, that can influence the events/parameters in the catalytic processes and catalytic reduction efficiency can be obtained through the investigation of these parameters via experimental studies. In this work, we have selected a few of the properties (transport, electrical and stability) and report their variations in the NF formulation with catalyst particle concentrations. Considering the multiple parameters that could be identified to influence the catalytic properties, in this work, we presume that the obtained values of these properties could be integrated in such a way to predict and control the kinetic/energetic bottlenecks of the specific properties/processes in the heterogeneous process. We believe that useful extrapolation would be possible to fine-tune the experimental conditions for achieving better catalytic efficiency for the reduction process.

**Figure 4:**
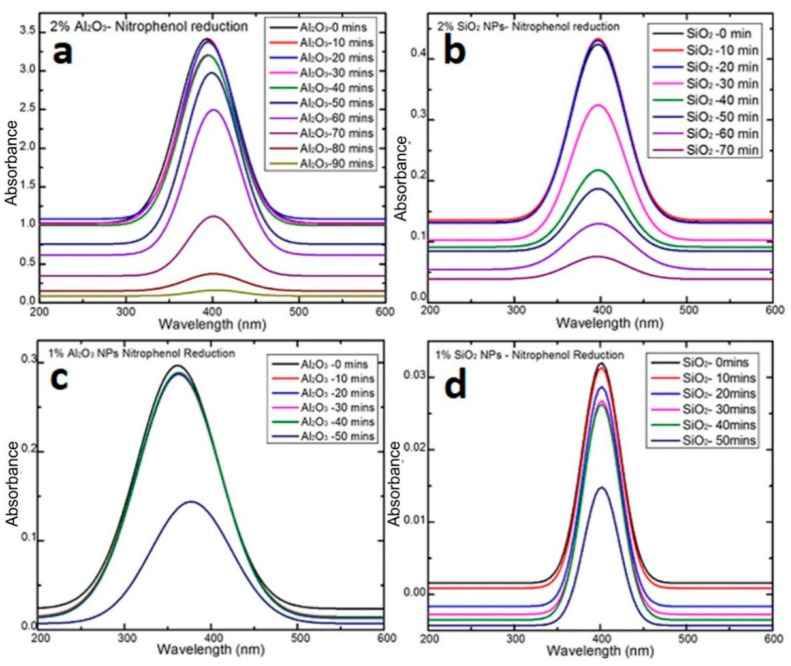
UV-Visible spectra recorded during the degradation of 4 NP in the presence silver added (**a**) 2% Al_2_O_3_ NF, (**b**) 2% SiO_2_ NF, (**c**) 1% Al_2_O_3_ NF and (**d**) 1% SiO_2_ NF.

In this study, the reduction of 4-NP in the presence of sodium borohydride (NaBH_4_) at ambient temperature was selected as a model reaction to assess the catalytic efficiency of Al_2_O_3_@Ag/SiO_2_ @Ag NFs. However, its reduction product, 4-aminophenol (4-AP), is less toxic than that of 4-NP [66]. Therefore, it has been considered that the efficient conversion of 4-NP to 4-AP by a facile and efficient catalytic method is of great importance. The conversion of 4-NP to 4-AP can be conveniently followed by UV-Visible spectroscopy. It is to be noted that the 4-NP aqueous solution has a distinct absorption peak at 315 nm. Upon the addition of NaBH4, the absorption peak shifts to 400 nm due to the formation of 4-nitrophenolate ions. We observed that there was no significant change in the absorbance of the 400 nm peak (absorbance of phenolate ions) over a long duration (more than 2 h) for simple Al_2_O_3_ and SiO_2_ NFs (1% and 2%) indicating the catalytic reduction of 4NP did not occur with these simple metal oxide-based NFs. The reduction of 4-NP was not observed in the absence of catalysts [67]. In this study, we examined the catalytic properties of Al_2_O_3_ and SiO_2_ NFs (1% and 2%) by exploiting the reduction of 4-NP with the addition of sodium borohydride. We learned that the yellow color of the solution was decolorized within certain minutes after the addition of Ag decorated Al_2_O_3_ and SiO_2_ NPs. The catalytic degradation of 4NP was followed by UV-Visible spectroscopy for every 10 minutes’ time interval over a period and shown in Figure 4 for 1% and 2% silver loaded Al_2_O_3_ and SiO_2_ NFs. The catalytic removal of 4-NP was monitored by UV–Vis spectroscopy at the characteristic peak at 400 nm, as presented in Figure 4. Both the Al_2_O_3_@Ag NF and SiO_2_ @Ag NF (1% and 2% weight) trigger the conversion of 4-NP, of course, with different speeds leading to a decrease of the absorption peak at 400 nm. This could be witnessed by the changes in the absorbance with varying extent overtime at 400 nm for these Al_2_O_3_@Ag NF and SiO_2_ @Ag NF (Figure 4).

**Figure 5:**
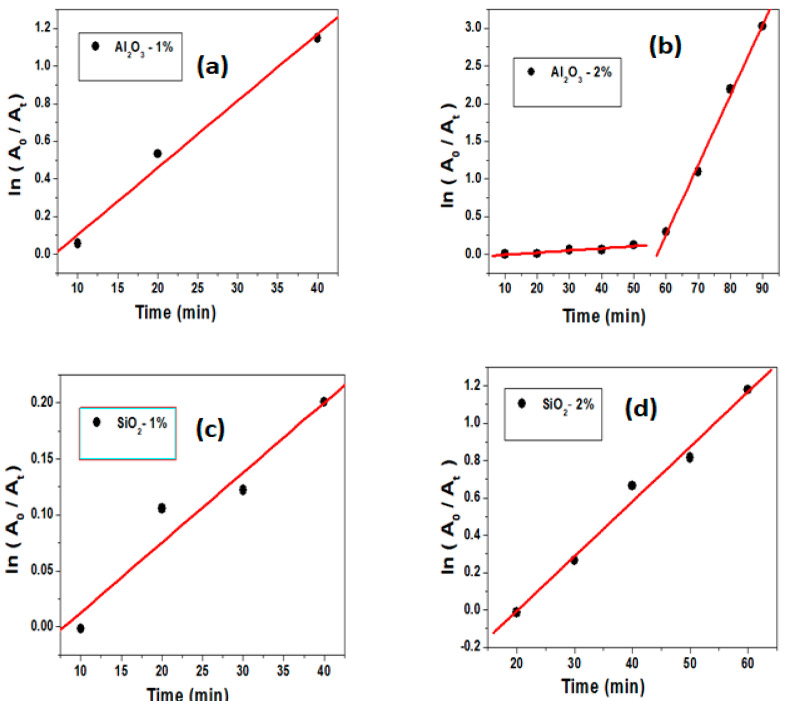
Kinetic plots of ln (A_0_/A_t_) versus time for the catalytic reduction of 4-NP in the presence of (**a**) 1% Al_2_O_3_ NF, (**b**) 2% Al_2_O_3_ NF, (**c**) 1% SiO_2_ NF and (**d**) 2% SiO_2_ NF.

Considering the excess amount of NaBH_4_ that was used as compared to 4-NP, the reaction kinetics is considered to follow the pseudo-first-order law following the kinetic equation as:

(5) $$-\ln\left(\frac{A_{t}}{A_{0}}\right)=-\ln\left(\frac{C_{t}}{C_{0}}\right)=k_{a_{\mathrm{PP}}}t$$

where k_app_ is the apparent kinetic rate constant, A_0_ and C_0_ represent the initial absorbance and concentration of 4-NP, A_t_ and C_t_ are the absorbance and concentration of 4-NP at time t, respectively. As noticed in Figure 5, there is a linear relationship between −ln (C_t_/C_0_) and t for the catalytic reduction of 4-NP in Al_2_O_3_@Ag NF and SiO_2_ @Ag NF (1% and 2% weight). We estimated the k_app_ from the slope of the linear plots in Figure 5 and presented in Table 5.

One could notice that the kinetic plot of catalytic reduction of 4-NP with 2% weight Al_2_O_3_@Ag based NF exhibited two linearity regions with two different slopes, inferring that the reaction follows slowly at the initial times (<60 min) and rapidly at a later time (>60 min). We noticed that increasing the weight % of Al_2_O_3_@Ag from 1% to 2% in the NF significantly enhances the k_app_. Typically, the k_app_ values of 1% to 2% Al_2_O_3_@Ag NF are 35.5 × 10^−3^ s^−1^and 92.9 × 10^−3^ s^−1^ (long time region) respectively. Similarly, one can notice that increasing the weight % of SiO_2_ @Ag from 1% to 2% in the NF greatly increases the k_app_. Typically, the k_app_ values of 1% to 2% SiO_2_ @Ag NF are 6.00 × 10^−3^ s^−1^ and 29.3 × 10^−3^ s^−1^. On comparing the catalytic capabilities of Al_2_O_3_@Ag NF and SiO_2_ @Ag NF, the former NF is more efficient for the 4-NP catalysis as compared to the latter NF. Importantly, the catalytic efficiency of 2% weight Al_2_O_3_@Ag NF for 4-NP removal is much higher than many of metal NPs based catalysts reported in literature: 55.3 × 10^−3^ s^−1^ [66] 25.1 × 10^−3^ s^−1^ [68] 25.1 × 10^−3^ s^−1^ [69] 9.58 × 10^−3^ s^−1^ [70] 9.95 × 10^−3^ s^−1^ [71] 80.19 × 10^−3^ s^−1^ [72] 80.19 × 10^−3^ s^−1^ [73] 32.7 × 10^−3^ s^−1^ [74]. We believe that the excellent stability of Al_2_O_3_@Ag NF (2% weight) as inferred from Zeta potential measurement is an added advantage for long-time use along with the high catalytic efficiency.

## 4. Conclusions

Our results have demonstrated that nanofluid formulations can be nanotechnologically advanced to accomplish the purpose of preserving environmental sustainability. Keeping this in mind, alumina and silica matrix were used as the catalytic support and their catalytic capabilities were enhanced upon appropriately loading with metal nanoparticles and suspended as a nanofluid formulation. The thermal properties of alumina and silica nanofluids were evaluated from the measured acoustic parameters. The variations in the activation energy and thermal conductivity of the two nanofluids were brought out and the reasons were discussed. Electrical properties like AC conductivity and dielectric constant of the NPs were also examined with varying a wide range of frequencies. The electrical conductivity of both the NFs is found to have a linear relation with the weight fraction of NPs and reduces gradually with temperature. Catalytic reduction properties of the silver added Al_2_O_3_ and SiO_2_ nanofluids were studied for the environmental application using a reduction of 4-nitrophenol as the model reaction. The catalytic capability of Al_2_O_3_@Ag NF is superior for the 4-NP catalysis as compared to the SiO_2_ @Ag NF. Interestingly, the nanofluid formulation shows superior catalytic performance than the simple catalytic dispersions reported in the literature. The present study on silver added NPs (Al_2_O_3_ and SiO_2_)-based NF for catalytic applications opens an avenue for further research with other NF formulations comprising metal oxide- metal nanoparticle combinations.

## Figures and Tables

**Table 1:** The relative viscosity of Al_2_O_3_ and SiO_2_ NFs.

Concentration (%)	Relative Viscosity (cP)							
	303 K		308 K		313 K		318 K	
	Al_2_O_3_	SiO_2_	Al_2_O_3_	SiO_2_	Al_2_O_3_	SiO_2_	Al_2_O_3_	SiO_2_
0.2	1.02	1.02	1.01	1.01	1.00	1.00	0.99	0.99
0.4	1.03	1.03	1.02	1.02	1.01	1.01	1.00	1.00
0.6	1.04	1.04	1.03	1.03	1.01	1.02	1.00	1.01
0.8	1.05	1.05	1.04	1.04	1.03	1.03	1.01	1.02
1.0	1.06	1.06	1.05	1.05	1.04	1.04	1.02	1.03
1.2	1.07	1.07	1.06	1.06	1.05	1.05	1.04	1.04
1.4	1.09	1.08	1.08	1.07	1.07	1.06	1.05	1.05
1.6	1.10	1.09	1.09	1.08	1.08	1.07	1.06	1.06
1.8	1.12	1.11	1.11	1.10	1.09	1.09	1.07	1.07
2.0	1.13	1.12	1.12	1.11	1.10	1.10	1.09	1.08

**Table 2:** Variations in electrical conductivity parameters of Al_2_O_3_ and SiO_2_ with applied frequency.

Frequency (Hz)	Impedance (Ω)	Angle (°)	Resistance (Ω)	Capacitance (F)	D-Factor (D)	Reactance (Ω)
	×10^6^	-	×10^6^	×10^−12^	-	×10^3^
100.00	6.61	11.18	6.74	46.30	5.1090	−3457.30
992.54	4.49	26.96	5.04	16.10	1.9740	−10,001.00
5704.02	2.21	49.00	3.39	9.51	0.8640	−2940.90
9851.50	1.60	56.25	2.89	8.41	0.6620	−1924.20
10,989.20	1.49	57.67	2.80	8.23	0.6270	−1762.70
1.08 × 10^6^	0.03	88.02	0.85	5.25	0.0320	−28.02
2.09 × 10^6^	0.02	91.60	−0.54	4.84	−0.0300	−15.78
3.23 × 10^6^	0.01	95.88	−0.12	4.16	−0.1060	−11.86
4.48 × 10^6^	0.01	101.10	−0.03	2.92	−0.3210	−12.18
5.00 × 10^6^	0.01	119.95	−0.02	2.23	−0.6039	−14.35

**Table 3:** Variation in electrical conductivity and pH of Al_2_O_3_ and SiO_2_ with weight fractions.

Weight Fraction of NPs in the NF	298 K (mS)		303 K (mS)		308 K (mS)		313 K (mS)		pH	
	Al_2_O_3_	SiO_2_	Al_2_O_3_	SiO_2_	Al_2_O_3_	SiO_2_	Al_2_O_3_	SiO_2_	Al_2_O_3_	SiO_2_
0.2	4.35	2.68	3.90	2.51	3.63	2.37	3.30	1.95	7.16	6.50
0.4	4.42	2.79	4.19	2.62	3.82	2.45	3.49	2.06	7.34	6.55
0.6	4.48	2.84	4.34	2.75	3.96	2.56	3.45	2.19	7.52	6.62
0.8	5.14	2.96	4.46	2.86	3.76	2.71	3.52	2.32	7.66	6.65
1.0	5.32	3.04	4.58	2.92	4.17	2.80	3.85	2.53	7.85	6.70
1.2	5.49	3.12	5.11	3.07	4.72	2.96	4.23	2.75	7.99	6.77
1.4	5.66	3.22	5.34	3.14	4.89	3.08	4.41	2.88	8.17	6.83
1.6	5.78	3.37	5.48	3.27	5.14	3.15	4.72	2.98	8.36	6.90
1.8	5.89	3.44	5.63	3.32	5.27	3.24	4.99	3.09	8.45	6.95
2.0	6.06	3.56	5.78	3.48	5.49	3.36	5.13	3.17	8.62	7.03

**Table 4:** Variation in ultrasonic velocity, density, and refractive index values of Al_2_O_3_ and SiO_2_ NFs.

Weight Fraction (%)	Velocity (m/s)		Density (kg/m^3^)		Ref. Index	
	Al_2_O_3_	SiO_2_	Al_2_O_3_	SiO_2_	Al_2_O_3_	SiO_2_
0.2	1485.0	1480.0	999.35	1003.20	1.335	1.337
0.4	1487.0	1482.0	1000.94	1003.70	1.337	1.338
0.6	1489.0	1484.0	1001.31	1004.40	1.338	1.339
0.8	1490.0	1485.5	1002.24	1005.50	1.339	1.340
1.0	1493.0	1487.0	1004.21	1006.50	1.340	1.341
1.2	1494.5	1489.0	1005.42	1007.60	1.341	1.342
1.4	1497.5	1491.0	1006.17	1008.60	1.342	1.343
1.6	1500.0	1493.0	1006.64	1009.40	1.343	1.344
1.8	1502.0	1494.5	1007.38	1010.10	1.344	1.345
2.0	1505.0	1496.5	1008.41	1011.80	1.345	1.346

**Table 5:** k_app_ values of Al_2_O_3_@Ag NF and SiO_2_ @Ag NF (1% and 2% weight fraction).

Nanofluids	k_app_ (×10^−3^ s^−1^)
Ag Al_2_O_3_-1%	35.5
Ag Al_2_O_3_-2%	2.8 (lower time region, <60 min)
	92.9 (Higher time region, >60 min)
Ag SiO_2_-1%	6.00
Ag SiO_2_-2%	29.3

## Data Availability

Not applicable.

## Abbreviations

Al_2_O_3_	Aluminum oxide
SiO_2_	Silicon dioxide
Ag	Silver
NPs	Nanoparticles
NFs	Nanofluids
Al_2_O_3_@Ag	Silver-loaded aluminium oxide
SiO_2_@Ag	Silver-loaded silicon dioxide
NaBH_4_	Sodium borohydride
4-NP	4-Nitrophenol

## Appendix A

### Materials Characterization

Distinctive results of microstructural and morphological characterization are presented and discussed. XRD patterns of Al_2_O_3_ and SiO_2_ NPs are shown in Figure A1 and Figure A2. The XRD pattern of Al_2_O_3_ consists of peaks correspond to the (012), (104), (202), (211), (214) and (217) of a rhombohedral structure that agrees well with the JCPDS, File No. (71-1683) (Figure A1). The XRD pattern of that SiO_2_ NPs (Figure A2) exhibits a hexagonal crystal structure with amorphous nature.

The average crystalline size was calculated using the Debye Scherer formula:

D = 𝑘𝜆/𝛽 𝑐𝑜𝑠 θ (A1)

where D is the crystallite size in nanometers, 𝜆 is the wavelength of the radiation (1.54056 A° for CuKα radiation), *k* is a constant equal to 0.94, 𝛽 is the peak width at half-maximum intensity, and θ is the peak position. The average crystallite size of Al_2_O_3_ is 1.5 nm.

Scanning electron microscopy (SEM) and transmission electron microscopy (TEM) analysis provide information on the morphology and particle size of the Al_2_O_3_ and SiO_2_ NPs, respectively. SEM image of Al_2_O_3_ NPs (Figure A3a) reveals the presence of irregular and agglomerated spherical particles. SEM image of SiO_2_ NPs (Figure A3b) reveals the presence of spherical particles with lesser agglomeration. TEM image of Al_2_O_3_ NPs (Figure A4a) reveals the particle size distribution and sizes of around 70 nm_._ TEM image of SiO_2_ NPs (Figure A4b) informs that the particles of sizes having the average size of 64 nm are distributed.

The energy dispersive X-ray analysis (Figure A5) confirms the purity of the metal oxides. The presence of the respective metal and oxygen elements only in the alumina and silica samples, as shown in Figure A5a,b suggests that there is no contamination.

SEM analysis along with energy-dispersive X-ray spectroscopy (EDS) provides information on the morphology, particle size and elemental composition of Ag loaded Al_2_O_3_ (Figure A6a–d). Figure A6a–c presents the SEM images which reveal the presence of randomly distributed spherical particles in the form of larger agglomerates with particle sizes ranging from ~40 to ~70 nm. EDS in Figure A6d reveals that the major elements are aluminum, oxygen and silver with weight % of 56.77, 42.24 and 0.29, respectively,

SEM images (Figure A6e,f,h) and EDS (Figure A6i) of Ag loaded SiO_2_ NPs inform the morphology, particle size and elemental composition of Ag loaded SiO_2_ NPs. SEM images (Figure A6f,h) reveal that the particles are predominantly spherical with particle sizes ranging from 34 nm to 47 nm. EDS (Figure A6i) reveals the elemental composition in weight % for oxygen, silicon and silver as 55.11, 44.61 and 0.29, respectively.
